# The Role of Healthcare Providers in Shared Decision Making for Cervical Cancer Prevention in Ghana: A Qualitative Study

**DOI:** 10.21203/rs.3.rs-5436571/v1

**Published:** 2024-12-13

**Authors:** Juliet Bonnah, Baffour Awuah, Michelle S. Williams

**Affiliations:** George Mason University; Komfo Anokye Teaching Hospital - Kumasi; George Mason University

**Keywords:** Cervical cancer, Healthcare provider, Shared decision-making, Screening, Vaccination, Recommendation

## Abstract

**Background:**

Cervical cancer is currently the second leading cause of cancer death among women in Ghana. Several studies have identified healthcare providers as key stakeholders in cervical cancer prevention. There is limited data on the role healthcare providers in Ghana play in shared decision making for cervical cancer prevention. The purpose of this study was to investigate healthcare providers’ perceptions about women’s cervical cancer prevention behaviors and their practices for recommending cervical cancer prevention services to their patients.

**Methods:**

In-depth interviews were conducted with healthcare providers (n=27) working at healthcare institutions in Ghana. Qualitative analysis was done using in-vivo coding and thematic content analysis. The PEN-3 model, and the SHARE approach were used as the theoretical framework for the study.

**Results:**

Healthcare providers were the main source of cervical cancer information for their patients. However, several providers acknowledged that they have inadequate knowledge about cervical cancer. Several providers stated that they seek their patients’ opinion about cervical cancer when recommending cervical cancer prevention services to them. Most of the providers recommend cervical cancer prevention services when patients have suggestive symptoms. They address their patients’ concerns and fears about cervical cancer by educating and reassuring them. The cost of preventive services, lack of knowledge, cultural and religious beliefs, were identified as major factors that influence their patients’ health seeking behavior for cervical cancer prevention services.

**Conclusion:**

In Ghana, healthcare providers play a significant role in cervical cancer prevention. Therefore, there is a need for training programs to empower providers in shared decision making, and in recommending cervical cancer prevention services to patients.

## BACKGROUND

Cervical cancer is the second leading cause of cancer death among Ghanaian women.[1] In Ghana, the majority of women with cervical cancer are diagnosed at late stages that are di cult to treat.[2–4] Cervical cancer screening tests can be used to detect precancerous lesions, making cervical cancer highly preventable.[5, 6] Cervical cancer is also treatable when it is detected at early stages.[7] There have been significant advances in cervical cancer prevention for low-resource settings. However, only a small percentage of Ghanaian women adhere to the World Health Organization’s (WHO) cervical cancer screening guidelines.[8, 9]

Previous studies have shown that sociocultural barriers to cervical cancer awareness among women in Ghana include lack of cervical cancer awareness stigmatizing beliefs about cervical cancer, and misconceptions about cervical cancer risk factors.[10–15] Culturally tailored interventions are effective at reducing sociocultural barriers to especially when they are combined with recommendations from healthcare providers.[8, 16, 17]

There is a lack of knowledge about the role of healthcare providers in shared decision making for cervical cancer screening among Ghanaian women. Understanding factors that impact healthcare providers recommendations for cervical cancer screening may aid in the development and implementation of interventions aimed at increasing adherence to the WHO cervical cancer screening guidelines.

The purpose of this study was to investigate healthcare providers’ perceptions about women’s cervical cancer prevention behaviors and their practices for recommending cervical cancer prevention services to their patients. Semi-structured interviews were conducted with healthcare providers in the Greater Accra Region. The PEN-3 Model and the SHARE Approach were used to guide the qualitative data analysis.[18, 19]

## METHODS

### Study Design, Sample, and Setting

The perception of women’s health seeking behavior and the recommendation practices of healthcare providers for cervical cancer prevention services among women in Ghana were explored using an exploratory descriptive qualitative approach. A purposive sampling strategy was used to recruit healthcare providers (n = 27) who were practicing in the Greater Accra Region of Ghana. Recruitment ended when data saturation was reached. In-depth interviews were conducted with eligible healthcare providers to explore their perceptions and practice of recommending cervical cancer prevention services to their patients.

### Participant Recruitment

A member of the research team, who is a Ghanaian healthcare provider, recruited participants using a snowball sampling method. Healthcare providers were eligible to participate if they were 18 years or older, currently work at a healthcare facility in the Greater Accra Region, and could speak, hear, and comprehend English or Twi.

### Data Collection

A semi-structured, in-depth interview was conducted with eligible healthcare providers via an online teleconference platform. At the beginning of the interview, the investigator obtained verbal informed consent from the participant. The participant also completed a web-based demographic survey. For each interview, participants answered questions that were asked by the investigator based on their perceptions and experiences in the clinical and community settings. The interviews lasted an average of 25 minutes. The interviews were audio recorded. The interview recordings were transcribed in English. Each participant received GHC100 ($9) as an honorarium after completing an interview.

### Constructs

The PEN-3 Model and the SHARE Approach were used as the theoretical framework for the semi-structured interview guide.[19, 20] The questions on the interview guide focused on the following constructs: (1) Recommendations for cervical cancer prevention Services: Participant’s tendency to recommend cervical cancer prevention services to their patients; (2) Perceptions about factors that influence patients’ cervical cancer prevention behaviors: Participant’s perceptions about the factors that influence women’s health-seeking behaviors with regards to cervical cancer prevention; (3) Shared decision-making: Participant’s role in helping their patients to explore options available to them for cervical cancer prevention and control; and (4) Cervical Cancer Knowledge: Participant’s knowledge about cervical cancer prevention and control. We also explored the participants suggested for strategies to improve shared decision making for cervical cancer prevention services in Ghana.

### Data Analysis

To ensure that the transcripts were accurate, the investigator who conducted the interviews verified all transcripts against the audio recording. Two members of the research team who are skilled in qualitative research analyzed the transcripts independently using Atlas.ti 24 software. The team members reviewed two of the coded transcripts to establish coder agreement. Inductive coding was first conducted by reading through the transcripts to identify concepts that emerged during the interview. A second round of coding was conducted to identify and organize the themes that emerged from the first round of coding according to the key theoretical constructs of interest. Finally, the research team members met to compare the similarities and differences in the analyzed data and to merge codebooks. The interpretation of the results was done after an agreement was reached. Demographic data was analyzed using SPSS v21 for Mac to calculate the mean and percentages of the demographic variables.

## RESULTS

### Demographics

The demographic characteristics of the participants are displayed in Table 1. The mean age of the providers (n=27) was 34.29 (±5.46) years. There was not a significant difference in the number of male participants (n=14, 52%) and female participants (n=13, 48%). Approximately 41% of the providers were Physician Assistants (n=11), making up the largest category of healthcare providers in our sample. About 52% of the providers (n=14) worked in a municipal hospital or a district hospital. The majority of the providers practiced internal medicine or general medicine (n=17, 60.71%).

### Cervical Cancer Prevention Services Within Healthcare Facilities

Among our participants, only one provider said that their facility had “good” rates of cervical cancer screening and the HPV (human papillomavirus) vaccination. The majority of the providers indicated that cervical cancer screening and HPV vaccination rates in their facilities were low. A few providers working in health centers and clinics stated that their facilities did not offer cervical cancer prevention services, consequently, they referred patients to district, regional, or tertiary hospitals to access those services.

### Recommendations for Cervical Cancer Prevention Services

#### Frequency of Recommendations

More than half of the providers stated that the frequency of recommending cervical cancer screening and/or HPV vaccination to their patients as a means of preventative care was low. Some providers said they recommend these services only during cervical cancer awareness month. Only a few providers said that they do not recommend these services at all. This is reflected in a participant’s statement below:

“In a year I should be looking at maybe six times. It’s not something I do very frequently, so maybe six times in a year. Yeah, I hardly do that…”

(Physician Assistant, Health Center)

#### Reasons for Recommendations

Very few providers mentioned that they recommend cervical cancer screening or the HPV vaccine as part of routine preventive care for all of their female patients. Some providers stated that they make recommendations for these services based on a patient’s age (e.g. being of reproductive age), sexual activity, or sexual history. Most of the providers stated that they recommend cervical cancer screening when patients have symptoms suggestive of cervical cancer. For example, a participant stated:

“Once I have the strong suspicion of maybe something happening to the cervix, I do recommend…”

(Physician Assistant, Health Center)

### Shared-Decision Making

When asked about what they say to their patients about cervical cancer, most providers said they address their patients’ fears or concerns about cervical cancer by educating. However, several providers were concerned about their patients’ lack of knowledge about cervical cancer. The majority of the providers said that they think patients do not get enough information to help them make decisions about cervical cancer prevention services.

When asked to discuss how they engage their patients in decision making about cervical cancer prevention services, most providers said that they seek their patients’ opinion or thoughts about cervical cancer when recommending prevention services to them. This is reflected in the quote below:

“Yeah, I seek their opinion because it’s patients right. Every person has the right for his or her care. So I seek the patient’s view. If there is something positive, that she can’t do it, I convince and educate her more on it. I don’t go further. Maybe I refer to the cervical cancer team for another second opinion to be given to her …”

(Midwife, Municipal Hospital)

#### Sources of Information

Friends, family, the internet, posters in the community, and provider referrals were the most rarely mentioned sources of cervical cancer information. Most participants believed that healthcare providers are the main source of cervical cancer information for patients. Other sources frequently mentioned included healthcare facilities, television, radio, and health talks at durbars (outreach programs) held in their communities. For example, one provider stated:

“OK, so with our setup, information is dispersed through the consulting room from practitioners or the public health unit, so most of the times it has to do with maybe durbar, where we go to do some general health talk…”

(Physician Assistant, Municipal Hospital)

### Provider Knowledge about Cervical Cancer

Nearly half of the providers said that they did not have adequate knowledge of cervical cancer or cervical cancer prevention services to make a diagnosis or recommendation for cervical cancer prevention services. Only a few providers mentioned that they had recently been trained to provide cervical cancer prevention services. Most of the providers said they had not received training on any aspect of cervical cancer prevention and control other than what they learned in school while they were completing their professional degree. The quote below illustrates the lack of cervical cancer knowledge among our participants:

“No, I wouldn’t say I have adequate knowledge because to be frank, I think after school that we learn about cervical cancer and then how it comes about and everything I’ve been practicing for almost ve years now and I’m yet to you suspect a case like that and even refer for further investigation, as at now I wouldn’t say I am much abreast with everything about cervical cancer because it’s not something I often meet on a daily basis…”

(Physician Assistant, Health Center)

### Factors that Influence Cervical Cancer Prevention Behaviors

The most common factors that influenced cervical cancer prevention behaviors, both positively and negatively were the cost of prevention services, knowledge about cervical cancer, and patients’ cultural and religious beliefs related to reproductive healthcare. The comments below are example of the providers’ beliefs about the influence of cultural and religious beliefs, and the cost of prevention services:

“…and then also the cost involved. After explaining all these things then you tell the person that going for the screening will cost like this, looking at the community that I find myself, they can hardly even afford this four square meals, and then you ask this person to go in for screening or the vaccine. The economic status of the person can either influence the person taking the screening or not…”

(Midwife, District hospital)

“OK, so in my setting one of the things I realize is about I should say their religion or their culture. In this setting they are more of traditionalists. There are more of people who believe in the herbal treatments. So most of the women get to the hospitals late or they prefer taking herbal treatments for this kind of sicknesses or infections, that is, even if they come. So it’s hard for them to actually come to the hospitals to even start complaining about whatever they are going through…”

(Physician Assistant, Health Center)

Additional influential factors mentioned by participants were peer advice for or against screening, having either a domineering partner or a supportive partner, an individual’s preference for alternative/herbal treatments when experiencing gynecological issues, and the stigmatization associated with cancer. These factors appeared to impact the providers’ decision-making practices. The comment below by one participant explains how a domineering partner could influence a woman’s behavior for cervical cancer prevention services.

“…then the other is also about the domineering nature of the men. OK. So most women feel that for them to even go and seek for health care, they should be allowed by their husbands, and this is something that is very di cult because most of the men do not see the need of the women seeking healthcare with this kind of problems…”

(Physician Assistant, Health Center)

Most of the providers mentioned that they consider the distance to the screening center, the age of the patient, the number sexual partners, patient’s financial status, patient’s educational status, existing underlying conditions, and the availability of vaccines and screening services when recommending cervical cancer prevention services to their patients.

### Strategies for Improving Shared Decision Making

The providers suggested several strategies for improving shared decision making regarding cervical cancer services. The majority of the suggested strategies that were targeted at healthcare providers focused on increasing provider’s knowledge about cervical cancer. In contrast, the most common strategies targeted towards patients focused on reducing structural barriers to cervical cancer prevention services. A summary of the suggested strategies and their potential impacts on providers and patients is displayed in Table 2.

## Discussion

We found that the lack of cervical cancer prevention services within their own healthcare facility was one of the main factors that affected the participants’ ability to provide recommendations for those services. In many LMICs, cervical cancer screening and the HPV vaccine are not commonly available at non-tertiary hospitals. For example, the results of studies done in Nigeria, Kenya, Malawi and Uganda also revealed that the lack of access to facilities for screening was a major barrier to cervical cancer screening [21–24]. Our participants perceived that several sociocultural and structural factors influence their patients use of cervical cancer preventions services. The factors discussed included the lack of awareness about cervical cancer, stigmatizing beliefs about cervical cancer, the use of traditional medicine to treat gynecological problems, and the cost of the services. These influential factors are aligned with the results of other studies that have focused on examining barriers and facilitators to cervical cancer screening in Ghana [8, 10, 11]. These perceived factors should be considered by providers when engaging patients in shared decision making. Malekzadeh et al., 2022 found that decision-aid-based counseling was effective at promoting cervical cancer screening. Tools such as that could be tailored to address a patients’ cultural beliefs about cervical cancer.

The results of our study revealed that cervical cancer knowledge was low among our participants. A previous study also found that there was lack of cervical cancer knowledge among nurses in rural and urban hospitals in Ghana.[15] This suggests that there is a need for the development and implementation of cervical cancer education training programs aimed at healthcare providers at non-tertiary hospitals. Previous studies have shown a healthcare provider’s recommendation for cancer screening combined with discussions about the recommendation have a positive impact on a patient’s decision to get screened.[25] Increasing providers’ knowledge about cervical cancer screening may increase their self-efficacy for engaging in shared decision-making about cervical cancer prevention services. Our participants also suggested that giving providers a reminder to recommend cervical cancer prevention services to patients would be helpful. Evidence from other studies have shown that provider reminders and the use of patient decision aids are effective at increasing cancer screening participation. [26–29] For example, Romero de Mello Sa et al. found that a reminder tool embedded in an electronic medical record was a cost-effective strategy for increasing female patients engagement in preventive health care [29]. Further research is needed to evaluate the use of interventions that can increase providers’ engagement in shared decision-making regarding cervical cancer prevention services.

### Implications

Signi cant advances have been made to make cervical cancer prevention services more accessible in LMICs. However, the results of our study indicate that additional efforts are needed to ensure that those services are available in all healthcare facilities, not just tertiary hospitals. In addition, there is a need for the development and implementation of training programs that will give healthcare providers the opportunity to develop the knowledge and skills needed to engage their patients in shared decision-making about cervical cancer prevention services.

### Limitations

A strength of our study was that we interviewed a variety of different types of healthcare providers. We also interviewed participants who worked at three levels of healthcare facilities. The diversity among our participants enabled us to get a range of perspectives about shared decision-making for cervical cancer prevention services. A limitation of our study was that all of our participants worked in the Greater Accra region of Ghana. The Greater Accra Region has the highest Human Development Index out of all 16 regions in Ghana. Therefore, our ndings may not be generalizable to other regions.

## Conclusion

We found that healthcare providers in the Greater Accra Region of Ghana did not frequently engage their female patients in shared decision-making regarding cervical cancer prevention. The lack of cervical cancer prevention services within healthcare facilities was one of the most common barriers reported. Limited knowledge about cervical cancer among the providers also contributed to low engagement in shared decision-making with their patients. Developing interventions to reduce modi able barriers will enable healthcare providers to actively engage their patients in shared decision-making about cervical cancer prevention services.

## Figures and Tables

**Table 1.** Demographic characteristics of the participants

Demographic Characteristic	n (%)
Age, mean ±SD	34.29 (±5.46)
	
Gender	
Female	13 (48%)
Male	14 (52%)
	
Marital Status	
Single	13 (48.15%)
Married	12 (44.44%)
Divorced	2 (7.41%)
	
Ethnic Group	
Akan	13 (48.15%)
Ewe	9 (33.33%)
Ga	4 (14.81%)
Foreigner	1 (3.70%)
	
Level of Healthcare Facility	
Municipal/District Hospital	14 (51.85%)
Health Center	10 (37.04%)
Polyclinic	3 (11.11%)
	
Type of Healthcare Provider	
Physician Assistant	11 (40.74%)
Physician	6 (22.22%)
Registered Nurse	5(18.52%)
Midwife	4 (14.81%)
	
Field of Practice	
Internal Medicine	17 (60.71%)
General Nursing	6 (21.43%)
Women’s Health	4 (14.29%)
Obstetrics and Gynecology	1 (3.57)
	
Age Range of Patients	
Both children and adults	20 (74.07%)
Adults 18 years or above	5 (18.52%)
Children under 18years	2 (7.41%)

**Table 2.** Healthcare Providers’ suggested strategies and the potential impacts

Suggested strategies	Potential Impact
**Target audience: Healthcare providers**	
Training programs Remind providers to recommend preventions services to patients	Increase provider knowledge on cervical cancer prevention to educate patients. Empower providers to suspect and diagnose cervical cancer and make appropriate recommendations. Enable provider to create awareness about cervical cancer.
**Target audience: Patients**	
Education Free or low-cost screening services Include cervical cancer screening in routine laboratory examinations.	Increase knowledge and awareness of cervical cancer. Decrease barriers to accessing cervical cancer prevention services.

## Data Availability

The data collected and analyzed during the study are not publicly available due to reasons of participant’s privacy and confidentiality but are available at the corresponding author’s institution and may be provided on reasonable request.

## Abbreviations

HPV: Human papillomavirus
